# Supplementary figures and images for: Structural foundation for the role of enterococcal PrgB in conjugation, biofilm formation, and virulence
Source: eLife. 2023 Oct 20;12:RP84427. doi: 10.7554/eLife.84427 (PMC10588982; doi:10.7554/eLife.84427)

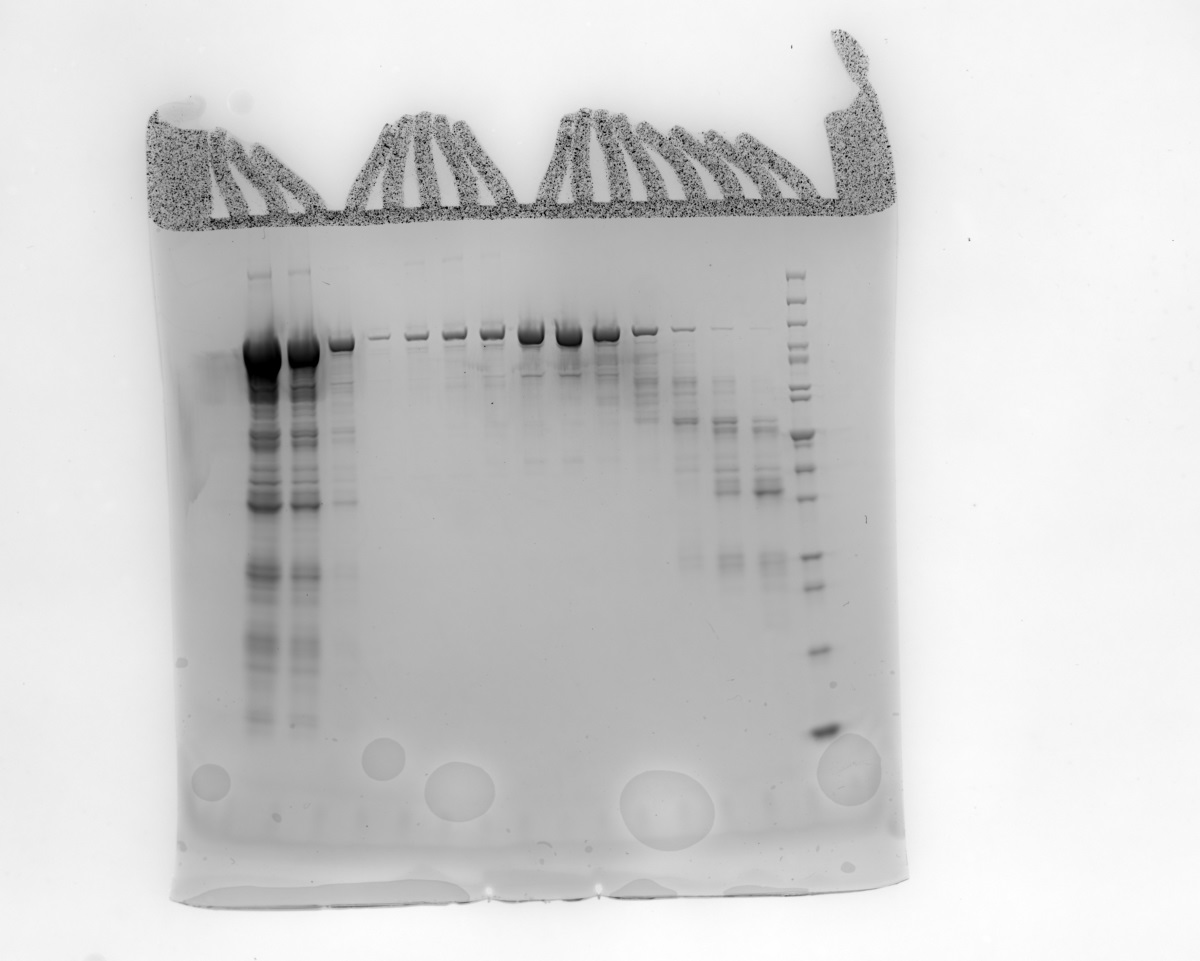

Supplement: Figure 1—figure supplement 1—source data 1. [file elife-84427-fig1-figsupp1-data1.zip › Fig 1 figure supplement 1 source data.tiff]

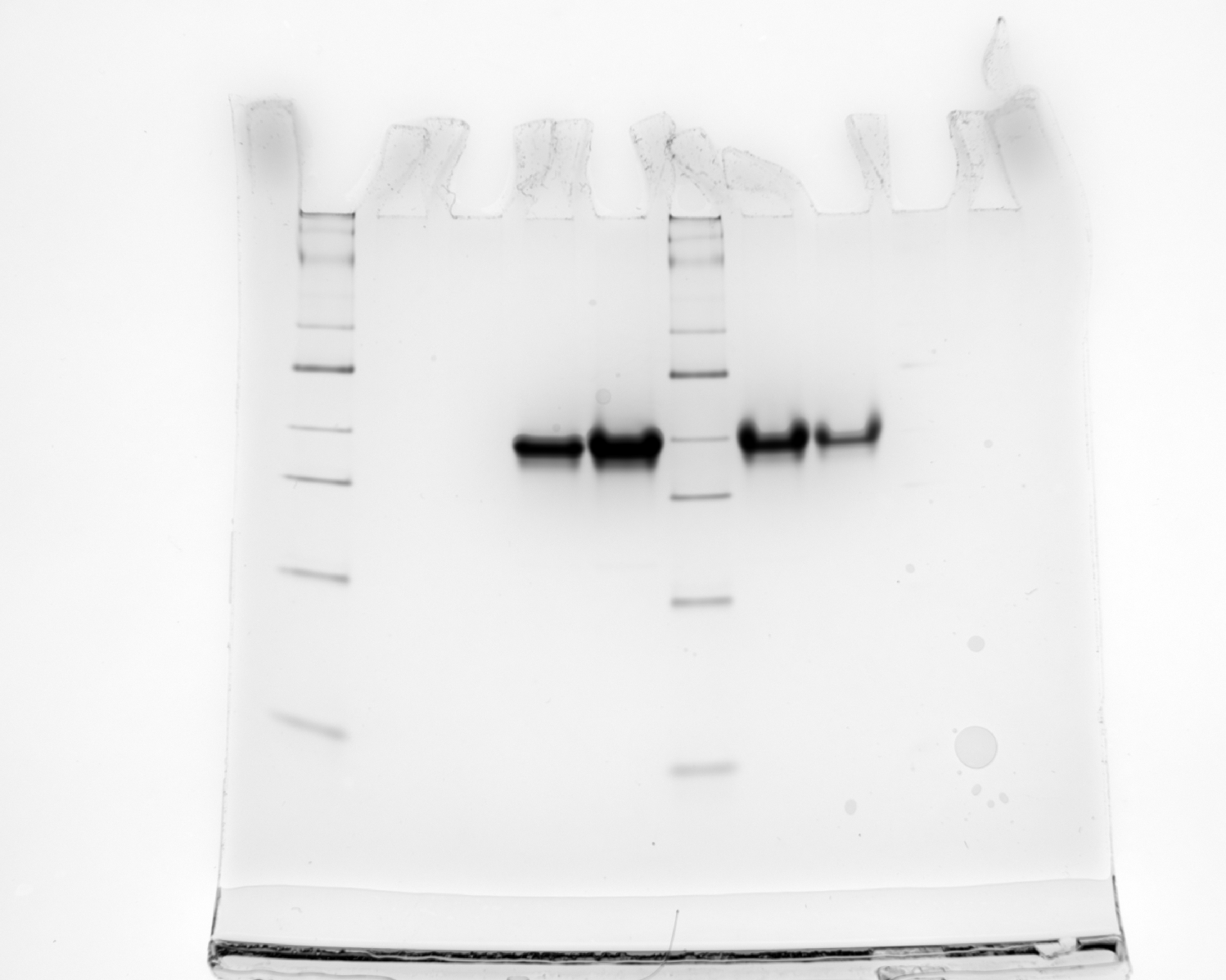

Supplement: Figure 2—figure supplement 4—source data 1. [file elife-84427-fig2-figsupp4-data1.zip › Fig 2-S4B native gel.tif]

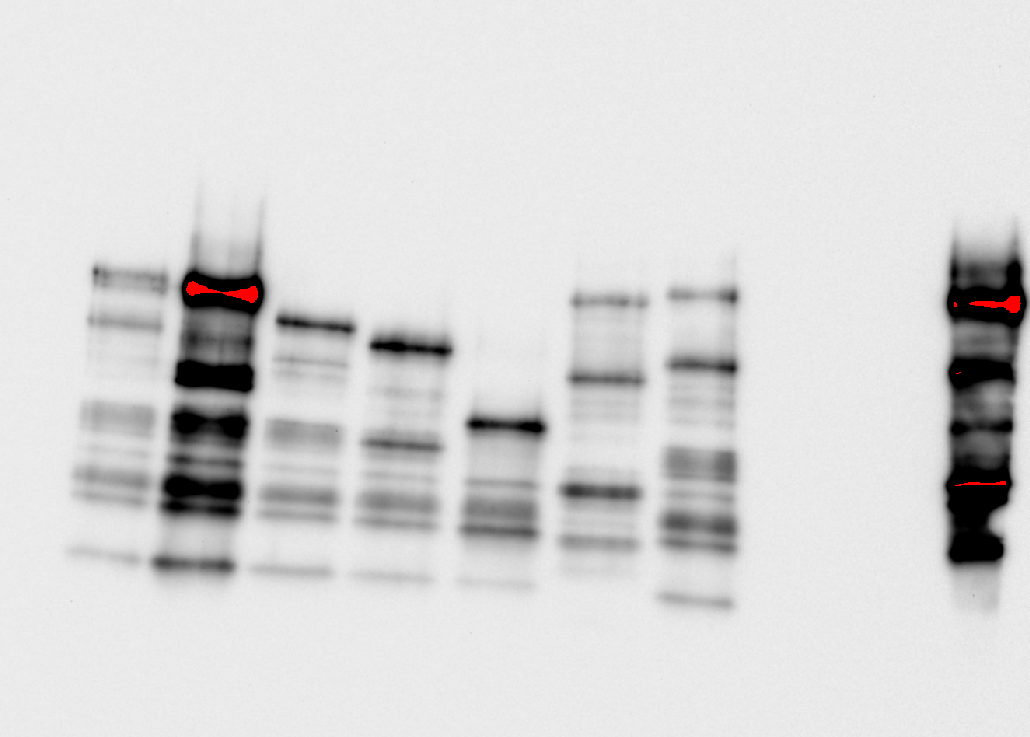

Supplement: Figure 3—figure supplement 1—source data 1. [file elife-84427-fig3-figsupp1-data1.zip › Fig 3 - supplementary figure 1A raw.tif]

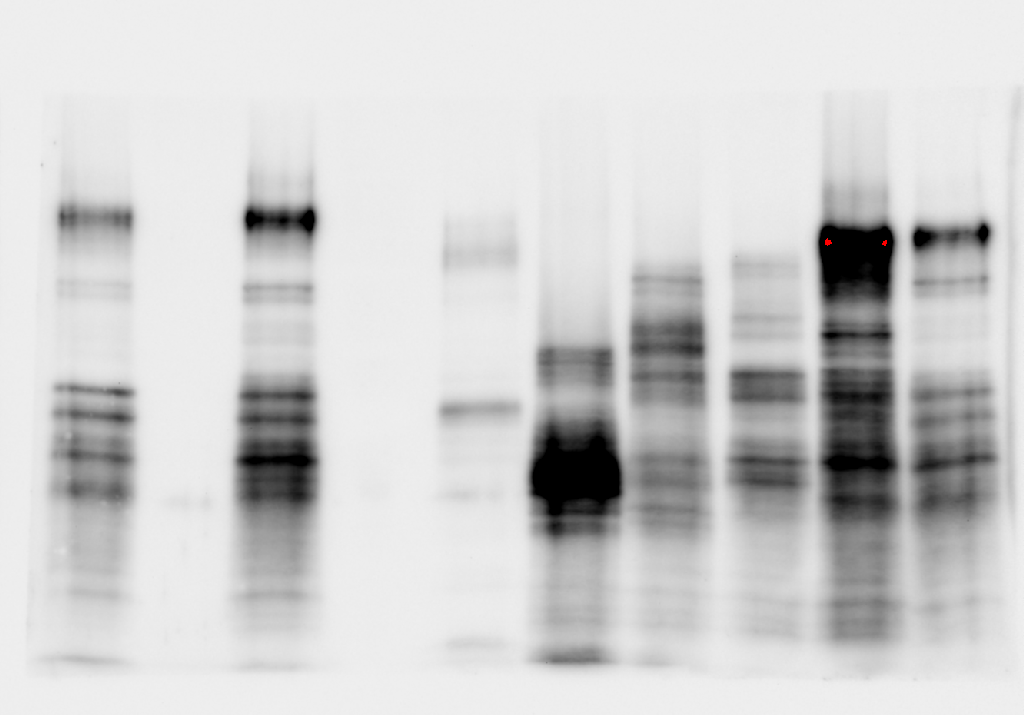

Supplement: Figure 3—figure supplement 1—source data 1. [file elife-84427-fig3-figsupp1-data1.zip › Figure 3 - supplementary figure 1B raw.tif]

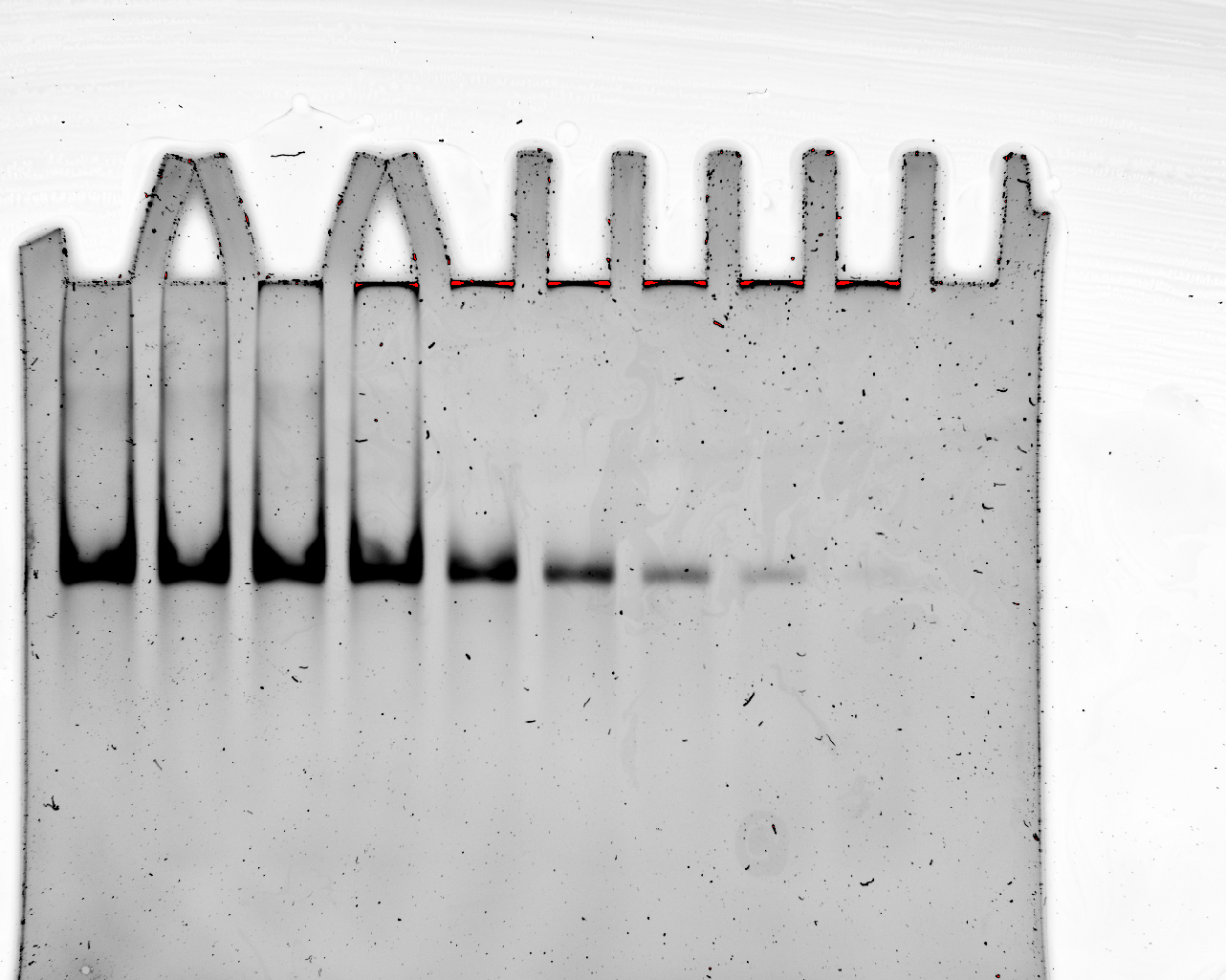

Supplement: Figure 4—figure supplement 1—source data 1. [file elife-84427-fig4-figsupp1-data1.zip › Fig 4 supplementary file 1A source data wt.tif]

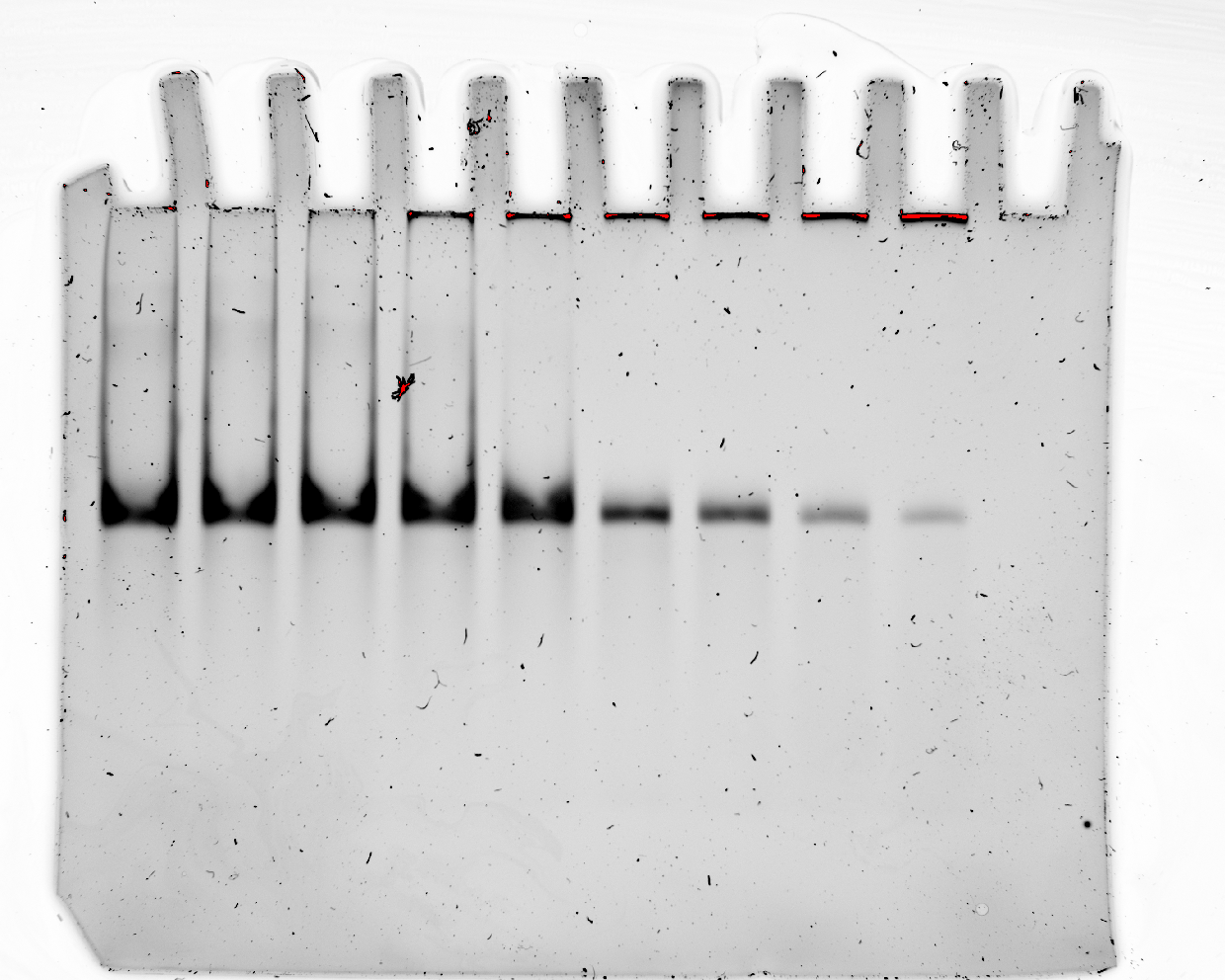

Supplement: Figure 4—figure supplement 1—source data 1. [file elife-84427-fig4-figsupp1-data1.zip › Fig 4 supplementary file 1A source data variant.tif]
